# A comparison of balance control during stance and gait in patients with inflammatory and non-inflammatory polyneuropathy

**DOI:** 10.1371/journal.pone.0191957

**Published:** 2018-02-23

**Authors:** Oliver Findling, Rens van der Logt, Krassen Nedeltchev, Lutz Achtnichts, John H. J. Allum

**Affiliations:** 1 Department of Neurology, Cantonal Hospital Aarau, Aarau, Switzerland; 2 Department of Neurology, Inselspital, University Hospital Bern and University of Bern, Bern, Switzerland; 3 Radboud University Nijmegen, Nijmegen, The Netherlands; 4 Division of Audiology and Neurootology, Department of ORL, University Hospital Basel, Basel, Switzerland; The Ohio State University, UNITED STATES

## Abstract

**Introduction:**

We compared changes in balance control due to chronic inflammatory demyelinating polyneuropathy (CIDP) and non-inflammatory (non-inf) polyneuropathy (PNP) to each other and with respect to healthy controls (HCs). Differences in patients’ subjective impressions of balance capabilities were also compared.

**Methods:**

Balance control of 11 CIDP patients (mean age 61.1±(sd) 11, 8 male) and 10 non-inf PNP patients (mean age 68.5±11.7, all male) was examined and compared to that of 18 age- and gender-matched healthy controls. Balance control during stance and gait tasks was measured as trunk sway angles and angular velocities with body-worn gyroscopes. Patients’ subjective impressions of balance were obtained using the Dizziness Handicap Inventory (DHI). The Neuropathy Impairment Score in the Lower Limbs (NIS-LL) was used to measure clinical disease status.

**Results:**

Non-inf PNP patients had slightly lower NIS-LL (13.5±7.2 vs. 17.9±15.1) and DHI scores (22.6±17.1 vs 27.6±16.3). Gait tasks showed a significant decrease in gait speed with respect to HCs for both patient groups but reduced trunk sway for non-inf PNP patients. Trunk sway during tandem walking and walking on the heels was greater for both groups than that of HCs. Sway during 2-legged stance tasks with eyes closed on a firm or foam surface was also greater than for HCs.

**Discussion:**

Compared to HCs both groups of patients have significantly greater sway for most stance and gait tasks accompanied by reduced gait speed. As for HCs, non-inf PNP patients reduced trunk sway with slower gait speed. In CIDP patients this compensatory strategy was absent, possibly due to a greater deficit of efferent and motor nerve fibers. An interpretation of these findings is that CIDP patients have reduced ability to decrease trunk sway with slower gait speed and is possibly associated with an increased risk of falls.

## Introduction

Polyneuropathy (PNP) is a generalized, relatively homogeneous disease process of the peripheral nerves. Usually the distal nerves are affected most prominently. In PNP the sensory proprioception of the lower legs (lower-leg proprioceptive loss (LLPL)) can be affected. As this sensory system plays a key role in balance control, PNP patients experience balance impairment during stance and gait [1, 2]. This balance impairment is one of the most prominent symptoms of PNP and is primarily a result of absent sensory responses from the lower legs [3, 4]. Without sensory input from the lower legs, balance corrections following perturbations to stance are still initiated in LLPL patients [3] but pathological instabilities in trunk sway are present [5]. These instabilities are also present in LLPL patients during quiet stance [2]. However it is not known if an equally unstable trunk sway is present during other standard clinical tests such as tandem walking or walking on the heels as well as during normal gait.

To maintain balance the central integration of proprioceptive information from the legs with other sensory information is necessary [6–9]. In polyneuropathy, because the afferent conduction of tactile and proprioceptive information is impaired, a deficit in stance balance control results and an increased risk of falling is assumed [10–12]. Furthermore, the amount of nerve involvement is correlated with quiet stance [1, 11] and perturbed stance instability [3, 4, 6], as well as the number of falls experienced [13]. However, depending on the type of peripheral neuropathy, different nerve structures are involved, possibly resulting in different types of impaired balance.

The prevalence of polyneuropathy has been reported to be between 2% and 7% with an increase to 13% in persons older than 80 years [14, 15]. Clinical symptoms include sensory and neuropathic symptoms, autonomic dysfunction and motor symptoms [14, 16–19]. Most polyneuropathies are length dependent and predominantly sensory [20, 21]. Common causes are diabetes, vitamin B12 deficiency, alcohol abuse or infectious reasons, although in approximately 25% of cases, the cause remains unclear (cryptogenic) [22, 23].

Chronic inflammatory demyelinating polyneuropathy (CIDP) is a rare autoimmune mediated variant of polyneuropathy affecting both sensory and motor fibers in both proximal and distal segments of all four limbs, that is in a more length independent way. The inflammation causes damage to the myelin sheaths leading to slowing or blocking the signal conduction [24]. The prevalence is approximately 0.003% [25]. CIDP can affect all ages and men are more affected than women [25]. The disease course can be progressive, relapsing-remitting or monophasic [24].

We performed this study to test the hypothesis that patients with length-dependent non-inflammatory (non-inf) PNP and length-independent CIDP would have different deficits in stance and gait balance control possibly due to the greater effect on motor fibres in CIDP. Furthermore, we wanted to determine if, for both groups, gait and stance balance control measured in the form of trunk sway, was equally greater than that of healthy controls. As far as we are aware, this is the first time such comparisons has been undertaken. The second objective of this study was to determine whether differences in balance changes between these two patient groups are related to the patients’ own perceptions of vertigo and as measured with the Dizziness Handicap Inventory (DHI) questionnaire.

## Materials and methods

### Subjects and balance tasks

For this study balance control was examined in 11 CIDP patients (mean age 61.1±11, 8 male) and 10 non-inflammatory (non-inf) PNP patients (mean age 68.5±11.7, all male). All patients were diagnosed for polyneuropathy by experienced neurologists accordingly current guidelines [17, 26, 27]. Diagnosis and classification of the neuropathy was established by electroneurography and electromyography tests carried out no less than 6 months prior to the current balance tests. Demographic details of the patients are provided in Table 1. All CIDP patients underwent treatment with immunosuppressive treatment or intravenous immunoglobulins or both at the time of the study. All CIDP patients had progressive neurological symptoms before initiating treatment but had stable disease status with residual symptoms over the last 6 months prior testing. Seven of the 10 non-inf PNP patients had cryptogenic PNP, 2 had diabetes, and 1 nephrotic PNP. Patients’ balance scores for stance and gait tasks were compared to those of 18 age- and gender-matched healthy controls whose data was reported in prior publications [28–30]. To clinically quantify the involvement of peripheral nerves we used the NIS-LL (Neuropathy Impairment Score in the Lower Limbs) [31] which was assessed by a neurologist 30 mins prior to the balance tests. The NIS-LL scale ranges from the normal value of 0, to the maximum of 88 with the absence of all motor, sensory and reflex activity in the lower limbs. It is applied bilaterally for each modality tested and is additive for all deficits. The NIS-LL includes an assessment of global sensory involvement of peripheral nerves with a maximum deficit score of 16. For this assessment light touch, pinprick and vibration are tested with well-established clinical procedures [32]. In addition, we assessed proprioception by using joint position matching which is also part of the NIS-LL. To assess motor deficits the NIS-LL protocol was also used. The motor tests assess 8 leg muscle groups bilaterally for muscle strength. The strength grading used for this ranges from 0 to 4 (paralysis), and includes intermediate scores (eg 3.5) Thus for a complete lack of muscle strength, a total score of 64 is possible. However, strength scoring is subjective and open to bias as it depends on the degree of muscle weakness as judged by the examiner. Reflexes are scored by measuring the reflex response at the quadriceps and triceps surae muscles. Reflexes are assessed as normal, decreased or absent, with a total score of 8 if all reflexes are absent. The clinical measures used in the NIS-LL have been shown to correlate well with electrophysiological testing and with examination repetition by another experienced neurologist within the same center [33].

**Table 1:** Patient Characteristics (means and standard deviations).

Demographics	CIDP Patients	Non-inf PNP Patients
Age in years	61.1 ±11.0	68.5 ± 11.7 ns
Male	8 of 11	10 of 10
Duration between diagnosis and balance tests (yrs)	2.6 ±3.0	1.8 ±2.7 ns
DHI score	27.6 ± 16.3	22.6 ± 17.1 ns
**Neurological Examination**		
NIS–LL score	17.9 ± 15.1	13.5 ± 7.2 ns
Motor NIS-LL score	11.2 ± 9.8	2.7 ± 4.0 *
**Gait and balance scores**		
Balance Control Index^a^ score	438.6 ± 186.9	407.6 ± 179.5 ns
Gait speed m/s	1.1± 0.2	1.0 ± 0.2 ns

* p = 0.023

The following abbreviations have been used: DHI—Dizziness Handicap inventory

NIS–LL—Neuropathy Impairment Score in the Lower Limbs; Motor NIS LL—Motor part of the Neuropathy Impairment Score in the Lower Limbs; ns–no significant difference (2-sided t test) between patient groups.

^a^The Balance Control Index is a composite score based on measures from several of the tests–see methods for details.

To obtain a subjective assessment of the patient’s balance problems we used the dizziness handicap inventory (DHI) questionnaire [34]. The DHI is a self-assessment tool to quantify the impact of dizziness on the patient’s everyday life. The questionnaire consists of 25 questions divided into 3 groups: functional, emotional, and physical. The answers to the questions are scored yes score 4, perhaps score 2 or no score 0. The total score ranges from 0, indicating no handicap, to 100, indicating a profound self-perceived handicap. DHI scores have been shown to increase with the frequency of a patient’s episodes of dizziness [34].

Exclusion criteria for all patients were the inability to walk without a walking aid and the presence of orthopedic problems or other diseases/disabilities e.g. a history of stroke, vestibular or extrapyramidal disorders that could affect balance. All subjects gave written informed consent in accordance with the Declaration of Helsinki prior to the experiments. The protocol was approved by the ethics committee of North-Central Switzerland (EKNZ), Approval No: 2015–096.

Balance of the patients was assessed by measuring trunk sway during a sequence of 12 stance and gait tasks. Based on previous data we have equated increased (with respect to HCs) values of trunk sway angle and angular velocity during gait with gait instability for two reasons. Firstly, because the sway is measured at lumbar 1–3 is close to the position of the center of body mass [35]. Secondly because in our work with elderly subjects [36, 37] as well as patients with Parkinson’s disease [30, 38] we have demonstrated that trunk sway angle and angular velocities outside of normal limits indicate a risk of falling. This conclusion is supported both on the records of the number of falls over the 6 months prior to testing elderly fallers and non-fallers, as well as an independent measure of risk of falling–the stops-walking-when-talking test [36].

All stance and gait tasks were performed in the same order by each patient and executed without shoes. The tasks used were chosen based on previous studies in our laboratory comparing balance for 12 stance and gait balance tasks between different patient groups and healthy controls [30, 39–41]. Trunk sway was measured with the SwayStar device (Balance International Innovations GmbH, Switzerland) which uses two gyroscopes to measure pitch (anterior-posterior) and roll (lateral) angular velocities of the lower trunk at a sample rate of 100 Hz. Angles were determined on-line by trapezoid integration of the velocity signals. The device is worn at the level of L3-L5 in the middle of the lower back of the patients near the body’s center of mass [30]. The SwayStar device has been validated by a number of patient studies, specifically those affected by multiple sclerosis and vestibular loss [30, 39–42].

Four 2-legged tests were performed with the feet spaced shoulder width apart. Two were performed with eyes open–on a normal surface and on a foam surface (height 10 cm, density 25 kg/m^3^)–and 2 eyes closed. Three 1-legged stance tasks were performed eyes open, two on a normal surface (right and left leg) and one on the foam surface. For the latter foam task, the patients were asked to use their better leg to stand on. The stance tasks were performed on foam to reduce the remaining contribution of lower-leg proprioceptive to balance control. Stance tasks were performed for 20 seconds or until the patient lost balance. The patients performed 4 walking tasks with eyes open, one with eyes closed: a tandem gait task which was performed by walking 8 tandem steps (W8tan); walking on heels for 3 meters (W3mheels); walking 3m while pitching the head up and down; walking 3 meters, eyes closed (W3mEC) and walking 8 meters, eyes open (W8mEO). Tasks were performed with eyes closed to eliminate visual inputs to balance control. For gait tasks, patients were asked to walk at their comfortable pace. At the beginning of each gait task the patients were asked to stand comfortably with feet hip-width apart to standardize the start of each test. Prior to the balance assessments patients filled out the Dizziness Handicap Inventory (DHI). The DHI is used clinically and in research to assess the impact of a person’s dizziness on their quality of life [34].

### Data processing and statistical analysis

The outcome measurements of each trial were 90% range and peak-to-peak roll angle range (RAR), pitch angle range (PAR), roll angular velocity range (RVR), pitch velocity range (PVR) for the complete trial and trial duration. The 90% range was determined using the histograms of pitch and roll angle and angular velocity having divided the peak to peak range into 40 bins. As there were few differences in significance for 90% and peak to peak ranges (see Tables 2 and 3), here we report the 90% ranges and associated statistics.

**Table 2:** Means of CIDP and non-inf PNP patients for 90% ranges and task durations of gait tasks.

	Roll Velocity (deg/sec)			Pitch Velocity (deg/sec)			Roll Angle (deg)			Pitch Angle (deg)			Duration (sec)		
	CIDP	non-inf PNP	p	CIDP	non-inf PNP	p	CIDP	non-inf PNP	p	CIDP	non-inf PNP	p	CIDP	non-inf PNP	p
Gait tasks															
w3mec (+/- SD)	33.40 (3.57)	24.15 (1.73)	ns	48.8 (3.99)	32.39 (4.05)	**0.017** **√**	4.79 (0.40)	3.70 (0.43)	ns	5.67 (0.60)	4.63 (0.55)	ns	7.88 (0.98)	7.85 (1.28)	ns
w8meo (+/- SD)	39.25 (3.45)	27.57 (1.07)	**0.014** **√**	60.79 (5.79)	38.70 (5.86)	**0.025** **√**	4.35 (0.40)	3.51 (0.30)	ns	5.85 (0.66)	5.01 (0.57)	ns	10.77 (0.92)	9.92 (0.94)	ns

**Table 3:** Means of all patients and healthy controls for 90% ranges and task durations of stance and gait tasks.

	Roll Velocity (deg/sec)			Pitch Velocity (deg/sec)			Roll Angle (deg)			Pitch Angle (deg)			Duration (sec)		
	healthy controls	all patients	p	healthy controls	all patients	p	healthy controls	all patients	p	healthy controls	all patients	p	healthy controls	all patients	p
**Two-legged- stance**															
s2eo (sem)	0.70 (0.07)	0.81 (0.09)	ns	1.61 (0.15)	2.29 (0.28)	ns	0.40 (0.06)	0.46 (0.54)	ns	1.16 (0.12)	1.43 (0.12)	ns	20.00 (0.00)	20.00 (0.00)	ns
s2ec (sem)	0.70 (0.08)	1.33 (0.30)	**0.024**	2.02 (0.25)	4.43 (0.96)	**0.001** **√**	0.52 (0.08)	0.61 (0.08)	ns	1.38 (0.11)	1.83 (0.24)	ns	20.00 (0.00)	20.00 (0.00)	ns
s2eof (sem)	1.44 (0.15)	3.42 (0.69)	**0.006** **√**	2.23 (0.23)	6.24 (1.55)	**0.001** **√**	0.88 (0.11)	1.53 (0.29)	ns	1.56 (0.17)	2.28 (0.38)	ns	20.26 (0.17)	19.66 (0.34)	**<0.000**
s2ecf (sem)	1.79 (0.21)	7.14 (1.68)	**<0.000**√	3.21 (0.34)	15.09 (3.62)	**<0.000√**	1.06 (0.13)	2.81 (0.63)	**0.005** **√**	2.19 (0.21)	5.09 (1.01)	**0.004** **√**	20.27 (0.18)	17.19 (1.24)	**<0.000**
**One-legged- stance**															
s1eo left (sem)	7.77 (1.61)	28.56 (3.97)	**<0.000√**	7.06 (1.13)	25.44 (3.59)	**<0.000** **√**	3.71 (0.81)	9.43 (1.53)	**0.001** **√**	3.00 (0.46)	8.08 (1.09)	**<0.000√**	17.38 (1.30)	7.53 (1.45)	**<0.000**
s1eo right (sem)	7.77 (1.61)	38.06 (5.25)	**<0.000√**	7.06 (1.13)	34.49 (6.22)	**<0.000√**	3.71 (0.81)	10.13 (1.27)	**<0.000** **√**	3.00 (0.46)	8.12 (1.18)	**0.001** **√**	17.38 (1.30)	7.36 (1.34)	**0.001**
s1eof (sem)	14.06 (3.30)	40.93 (5.02)	**<0.000√**	8.83 (1.22)	29.80 (4.10)	**<0.000√**	5.19 (1.078	14.36 (1.84)	**<0.000√**	3.66 (0.56)	8.02 (0.99)	**0.001** **√**	16.35 (1.40)	9.53 (1.52)	**0.001**
**Gait tasks**															
w8tan (sem)	22.07 (2.06)	35.99 (2.96)	**0.002** **√**	25.72 (2.67)	42.15 (3.29)	**0.001** **√**	6.16 (0.95)	11.32 (1.27)	**0.001** **√**	6.01 (0.55)	11.17 (0.93)	**<0.000√**	10.81 (0.78)	12.60 (0.88)	ns
w3m heels (sem)	31.50 (1.69)	42.44 (2.94)	**0.012** **√**	36.47 (2.75)	59.05 (4.40)	**<0.000√**	3.90 (0.28)	7.23 (0.60)	**<0.000√**	5.30 (0.31)	9.64 (1.04)	**<0.000√**	6.77 (0.42)	11.86 (1.20)	**0.001**
w3mhp (sem)	25.27 (1.59)	27.48 (1.88)	ns	33.89 (2.43)	42.92 (4.98)	ns	4.37 (0.29)	4.56 (0.43)	ns	7.04 (0.63)	5.89 (0.46)	ns	5.54 (0.32)	7.24 (0.52)	**0.013**
w3mec (sem)	24.44 (2.97)	29.24 (2.32)	**0.014**	26.28 (1.70)	41.42 (3.35)	**0.001** **√**	4.38 (0.39)	4.30 (0.31)	ns	5.76 (0.46)	5.20 (0.42)	ns	4.74 (0.25)	7.87 (0.77)	**0.001**
w8meo (sem)	44.01 (3.54)	33.99 (2.33)	**0.021**	55.39 (10.61)	50.85 (4.75)	ns	4.94 (0.70)	3.97 (0.27)	**0.041**	7.88 (0.60)	4.47 (0.44)	**0.003**	6.93 (0.33)	10.34 (6.65)	**<0.000**

The following abbreviations have been used: s—standing, 2—two legs, 1- one leg, eo—eyes open, ec—eyes closed, f—foam support, w -walking, 3m – 3 metres, 8m – 8 metres, heels–on heels, 8tan– 8 tandem steps, hp–head pitching, ns–not significant (p>0.05), sem–standard error of mean.

√ means significant also for peak to peak range

We concentrated on 3 primary measures to compare between the groups. Firstly, a global balance control index (BCI) which combines results from several different trials into one index (see details below), secondly trunk sway measures for walking eyes open and closed which we hypothesised would be different from HCs, and thirdly trunk velocity measures during stance eyes closed which we expected to differ from those of HCs based on prior research [2]. The remaining tests explored were basically typical clinical tests–tandem walking and walking on the heels. The BCI is an additive composite score based on measures from several tests: From the test standing on 2 legs on foam with eyes closed (2*pitch velocity), for walking 8 tandem steps (1*roll angle), for walking 3m eyes closed (1.5*pitch velocity + 20*duration), walking 3m while pitching the head up and down (1.5*pitch velocity) [28]. The step-wise discriminant analysis used to select the above tests and measures entering the BCI is described in Allum and Adkin, 2003 [43]. The upper 95% limit of this index for healthy persons of the age of the patients is 340 [28]. This combination of the selected balance outcome measures has been shown previously to have a high accuracy in detecting patients with impaired balance [43].

To compare the PNP and CIDP patients, ANOVAs were performed followed by post-hoc t-tests. As statistical significant differences between these patient groups were only found for the walking 8m eyes open and 3m eyes closed gait, we also pooled the patient data for comparisons with health controls (HCs) using ANOVA tests. Comparisons with healthy age- and gender-matched normal values and between patient populations were performed post-hoc using independent-samples t-tests with a Bonferroni correction for 5 comparisons per balance test. Significance was set at p<0.05 after the Bonferroni correction.

## Results

### Differences between non-inf PNP and CIDP patients

**Fig 1:**
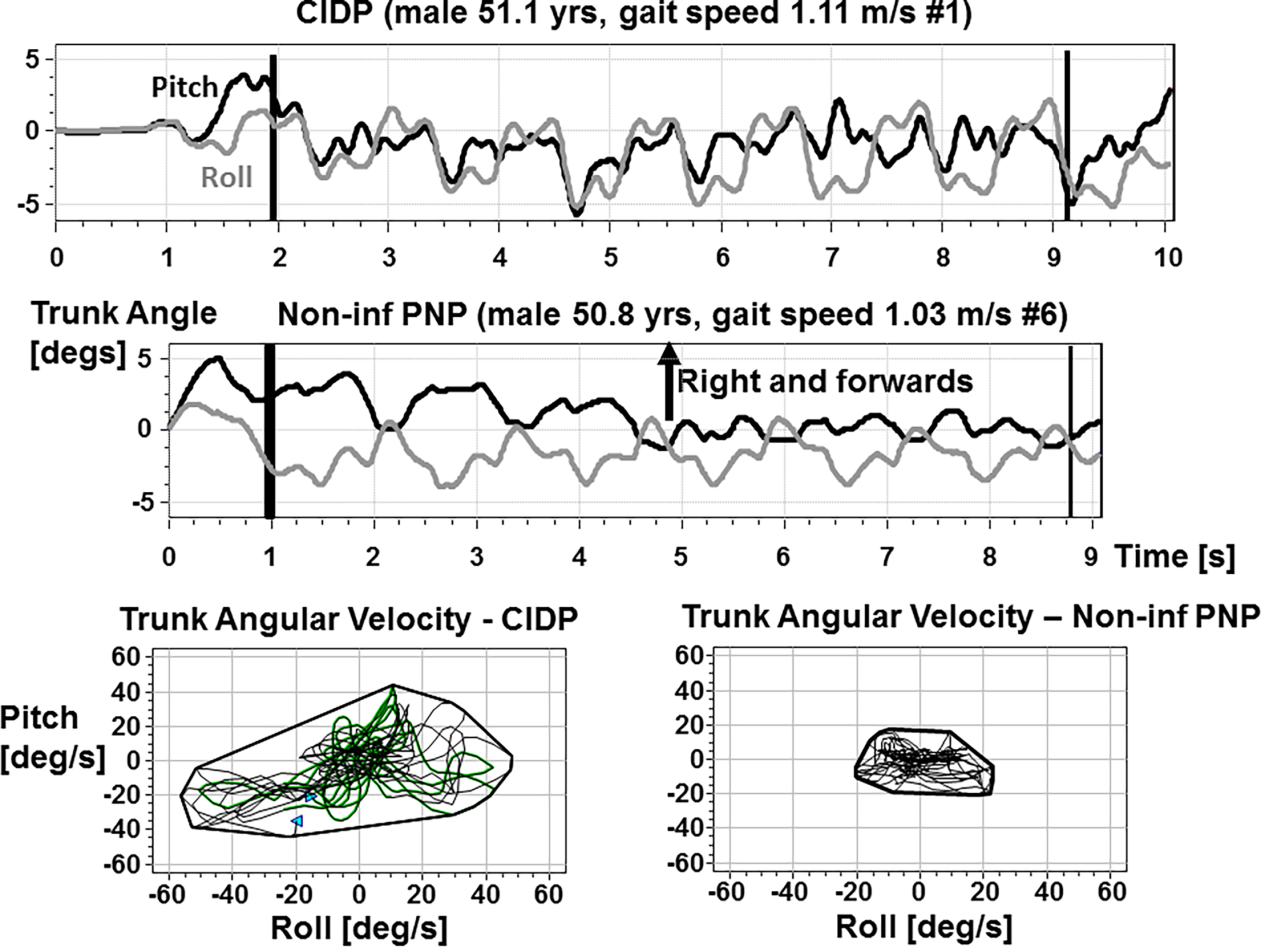
Examples of pitch and roll trunk sway during walking 8m with eyes open for a typical CIDP and a non-inf PNP patient. The upper traces are the time plots with the 8m measurement sequence marked with vertical lines indicating when the subject passed light barriers spaced 8m apart. The lower x-y plots are of pitch versus roll velocity over the 8m walking interval. The envelope of the angular velocity excursions is presented as a convex hull around each x-y plot. As indicated, both subjects are of the same age and walk with similar gait speeds. Note the larger trunk velocity for the CIDP patient.

**Fig 2:**
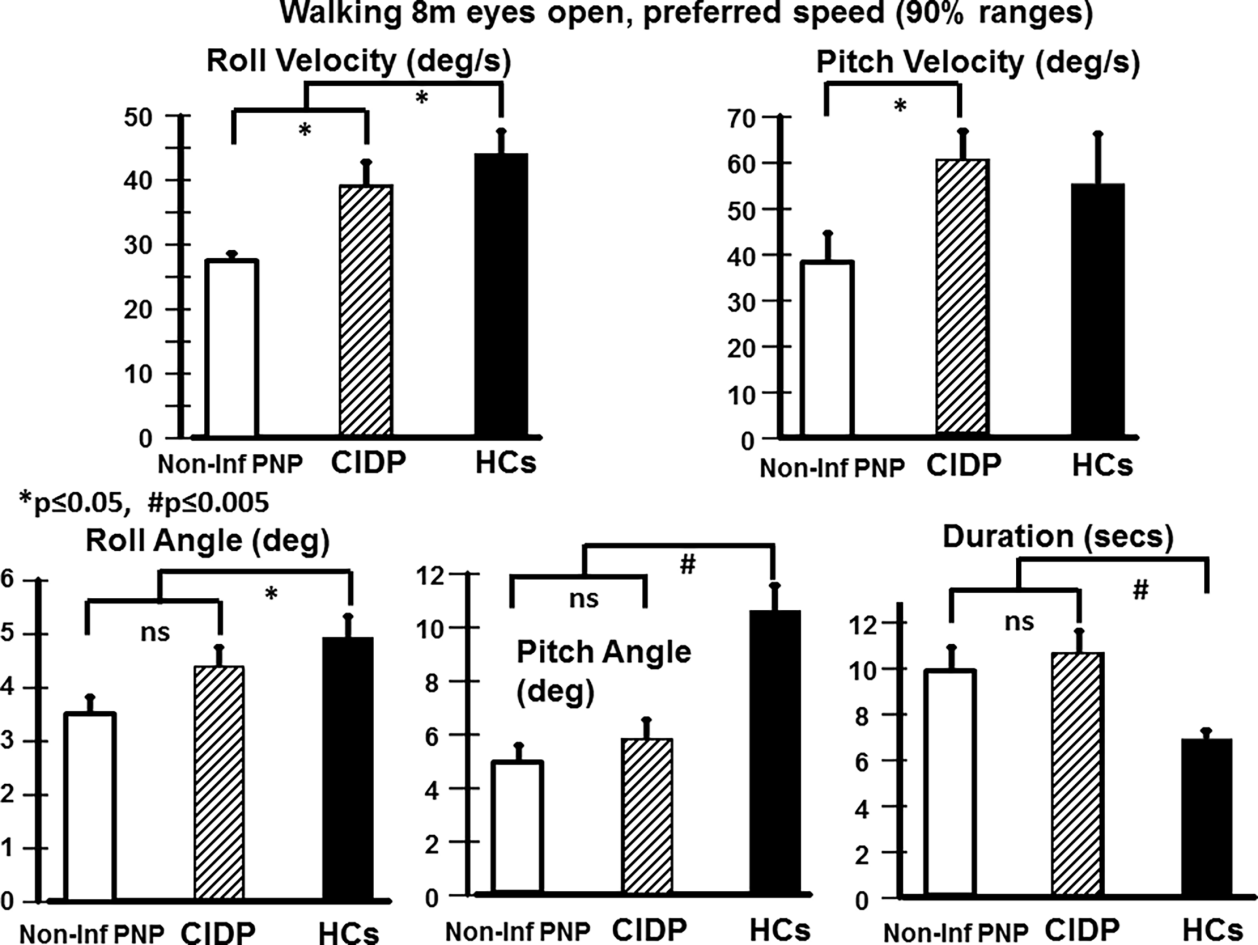
Mean 90% trunk sway measures for walking 8m with eyes open for trunk sway pitch and roll angle and angular velocity. The height of the column represents the mean value and the vertical bar above the column the standard error of the mean (sem). Significance is shown in the figure between the means of the non-inf PNP and CIDP patients, and between the pooled mean of the patients and that of the healthy controls (HCs). The concomitant reduction of sway velocity observed in our non-inf PNP patients has been observed previously in healthy elderly controls when they reduce gait speed. Note that sway velocity for the CIDP patients is not less than that of HCs despite the markedly reduced gait speed (walking duration over 8m is significantly greater for the patients).

**Fig 3:**
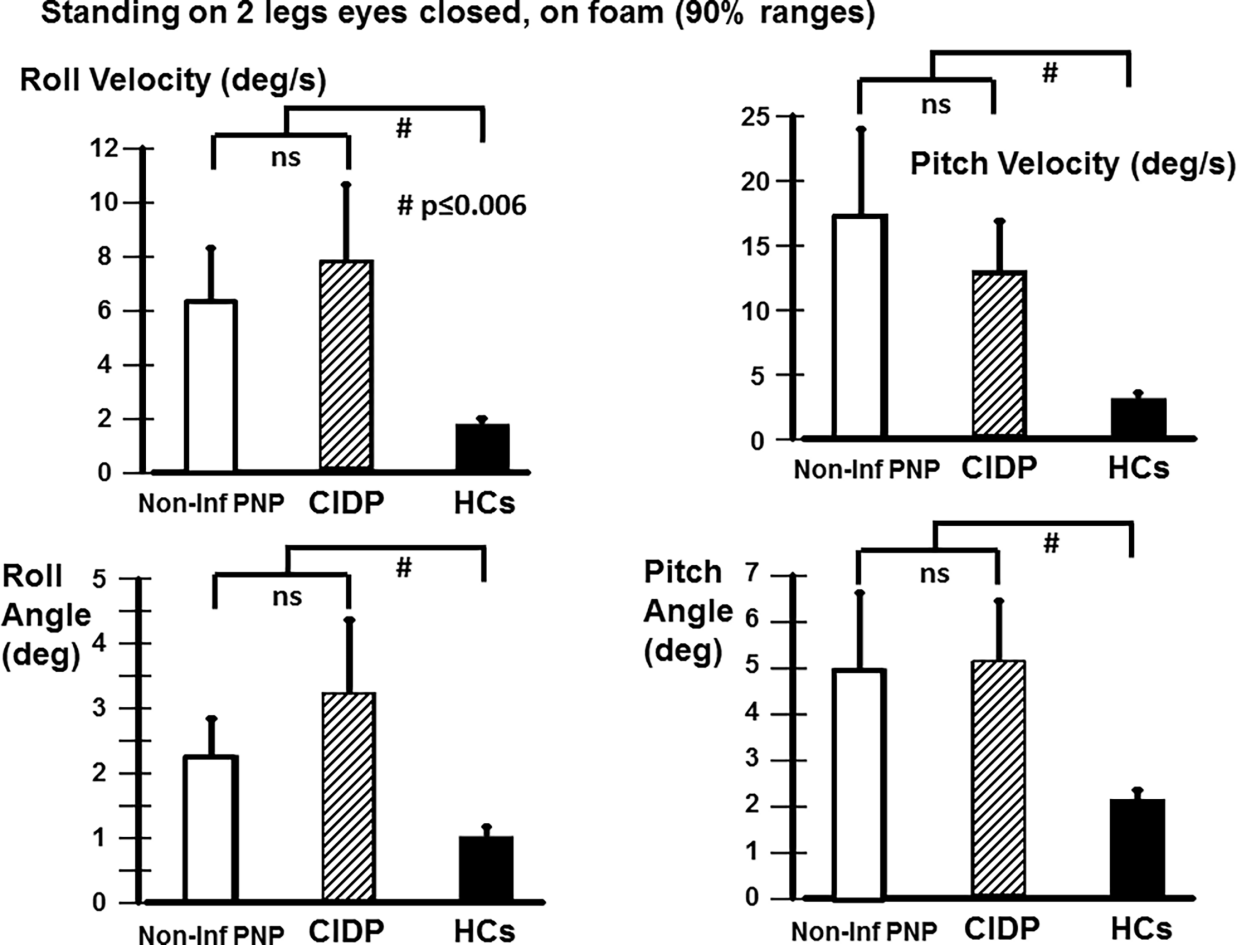
Mean trunk sway measures for standing on two legs eyes closed on a foam surface. The layout of the figure is identical to that of Fig 2. Note that all sway measures showed highly significant increases (p≤0.006) with respect to HCs.

As Table 1 indicates, there were no statistical differences between most of the 2 groups of patients’ measures even though the DHI, NIS-LL and BCI measures were slightly higher for the CIDP compared to non-inf PNP patients and the motor part of the NIS-LL revealed significantly higher scores for the CIDP patients. Thus our first primary measure (BCI) was not different between the two patient groups. There was, however, a major difference in trunk sway measures between the groups for normal walking with eyes open and closed (see Figs 1 and 2, and Table 2). This difference in our second primary measure is illustrated for eyes open walking in Figs 1 and 2. Both the non-inf PNP and CIDP patients have reduced gait speed compared to the approximately 7 seconds HCs required on average to walk 8m at their normal walking speed. However, in contrast to HCs [29] and the non-inf PNP patients (Fig 2), CIDP patients did not have reduced trunk sway velocities with slower gait speed. A similar effect was noted for pitch velocity walking eyes closed. No other differences between the two patient populations were noted in the balance control measures for the stance and other gait tasks even though CIDP patients tended to have large roll sway across all tests (see, for example, Fig 3). Due to the lack of a large number of patient group differences, we pooled the patient data when comparing the patient data with that of HCs (see Table 3 and Figs 2 and 3).

### Differences between healthy controls and patients

For the two-legged stance tests standing eyes closed on a normal and on a foam surface, the patients had significantly greater trunk sway velocities than HCs (Table 3). Thus our second and third primary measures, respectively gait and stance eyes closed were significantly different from HCs. This difference to HCs was also observed for trunk sway velocities eyes open on a foam surface (Table 3). Significant increases in sway angles with respect to HCs were also present for standing eyes closed on foam (Table 3 and Fig 3). Generally, sway for two-legged tests tended to be larger for CIDP compared to non-inf PNP patients but, as mentioned above, this difference was not significant.

As expected from the results with two-legged tasks, one-legged eyes open stance tests also showed highly significant differences to HCs. Table 3 indicates that the duration of one-legged stance was considerably shorter than that of HCs. The shorter duration was often accompanied by large sway deviations at the end of recording as the patients lost balance control.

For common gait tests used in neurology clinics to test patients (walking on the heels and walking tandem steps) significantly larger sway angles and velocities differences were also observed (Table 3).

Across all tests there was little difference between using peak-to-peak or 90% range measures. Peak-to-peak measures led to a slightly lower number of significant findings (28 versus 33 sway measures).

## Discussion

In this study we performed stance and gait posturography on patients with non-inflammatory polyneuropathy (non-inf PNP) and CIDP in order to investigate differences in trunk sway between the patient groups. As there is evidence that patients with polyneuropathy in general have trunk instabilities during stance tests [2], we also investigated whether patients with polyneuropathy have trunk instability (larger values than HCs) also during complex gait tasks and normal walking.

We found that both CIDP and non-inf PNP patients have significantly greater sway than HCs during stance tests and complex gait tasks (walking on the heels and walking tandem steps). (Fig 3, Table 3). In these tasks no significant differences have been found between non-inf PNP patients and patients with CIDP. However, while walking normally with eyes open and walking with eyes closed non-inf PNP and CIDP patients had slower preferred walking speeds (Fig 2). In non-inf PNP patients compared to CIDP patients we found clearly reduced trunk sway in addition to the slowing of gait (Fig 2). Interestingly, reduced trunk sway velocities have been reported in healthy controls when they slow their gait and we assume that this is a mechanism to increase trunk stability [29]. As the reduction of trunk sway velocity is associated with a reduced risk of falls in the elderly [36, 37], it would be interesting to investigate whether the reduction of gait speed in non-inf PNP patients is an attempt to improve overall gait stability by reducing trunk sway velocity. Based on this assumption, for CIDP patients the lack of a reduction of sway velocities despite a reduction of gait speed (Figs 1 and 2) may be an expression of a gait deficit due to the higher extent of nerve fiber involvement in CIDP. While the majority of non-inf PNP deficits are length dependent and sensory predominant, in CIDP sensory and motor fibers of the arms and legs are involved [21, 44, 45]. In contrast to pure sensory involvement with impaired afferent conduction of tactile and proprioceptive information in non-inf PNP, the additional motor involvement in CIDP may lead to impaired motor responses. Our CIDP patients had noticeably increased (weaker) motor scores (Table 1). It is known that distal muscle weakness has a more profound effect on balance control than proximal muscle weakness [35] which could explain our findings in CIDP patients. There is evidence that in patients with motor neuropathy the recruitment of lower limb muscles is delayed resulting in decreased stability during gait [13, 46, 47]. There is a clear proportionally increasing relationship between walking speed and trunk sway velocity in the elderly [29]. This effect may be more prominent during normal walking than during complex gait tasks such as walking while pitching the head up and down, which are usually performed with lower speed and less counterbalancing arm movements. It should be noted that the effect we observed for CIDP patients cannot be explained by any age effect as the CIDP patients were slightly younger than the non-inf PNP patients (61 vs 68 years on average). Sway increases with age after 60 years in healthy controls [28, 29]. Furthermore, as all CIDP patients had stable disease state at the time of the study, the behavior of CIDP (relapsing-remitting vs. progressive) had no influence of the results.

During the gait task walking 8m both groups of PNP patients slowed gait presumably in order to reduce trunk sway to within normal stability (trunk sway) bounds for that gait speed [29]. Our findings indicate that the CIDP patients did not reduce sway to amplitudes expected for their slower walking. In addition, amplitudes for stance tasks are increased in both groups of PNP patients. The question arises whether these results imply, particularly for the CIDP patients, an increased risk of falls in these patients. An association between sway angle and angular velocity amplitudes and the risk of falls has been shown in earlier studies with elderly persons as well as in patients with Parkinson’s disease [36–38].

Given the contribution of both vestibular and lower leg proprioceptive inputs to postural control [2–4] it is interesting to compare the effects of the two types of loss on balance control. Our results during stance with a greater number of PNP patients confirm previous trends [2] that 2-legged stance tests with eyes closed on a normal or foam surface or eyes open on foam are pathological in lower leg proprioceptive loss (LLPL) patients whereas as only standing eyes closed on foam is pathological for bilateral peripheral vestibular loss (BVL) patients [2] but not pathological for chronic unilateral vestibular loss patients [43]. Common to both types of sensory loss is the inability to walk within normal trunk stability bounds with eyes closed (see Table 3 for LLPL and Allum et al [48] for unilateral vestibular loss patients). However, apart from reduced durations more complex gait tasks (such as walking with head-pitching movement) are performed normally (Table 3 and Allum et al [48]) by both vestibular and lower leg proprioceptive loss patients. Thus, it appears both types of sensory inputs are equally important during gait, with leg proprioceptive inputs more important during stance.

In conclusion, in addition to unstable stance eyes closed, we found that patients with CIDP and patients with non-inf PNP have slower preferred gait speed than healthy persons. However, patients with non-inf PNP showed a reduction of trunk sway velocity while slowing gait which is similar to HCs when they slow their gait [29]. In contrast, in patients with CIDP we found a pattern different from HCs with no reduction of trunk sway despite reduced gait speed. Whether our findings are the expression of more severe balance deficits in patients with CIDP and whether their reduction of sway while slowing gait is part of an attempt to improve overall gait stability remains unclear. An expanded investigation of overall gait stability at different gait speeds would appear to be necessary to identify those PNP patient groups with a higher risk of falling and offer them an appropriate physiotherapy.

## Supporting information

S1 Table (XLSX)

S2 Table Information ANONnormal group. (XLSX)

S3 Table LLPL CIDP 1–11 ANONAarau Orig values. (XLSX)

S4 Table LLPL PNP 1–10 ANONAarau Orig values. (XLSX)

S5 Table Patients LLPL study ANON. (XLSX)

## References

[1] Boucher P, Teasdale N, Courtemanche R, Bard C, Fleury M. Postural stability in diabetic polyneuropathy. Diabetes Care 1995;18:638–45. 8586001 10.2337/diacare.18.5.638

[2] Horlings CG, Kung UM, Bloem BR, Honegger F, Van Alfen N, Van Engelen BG, et al Identifying deficits in balance control following vestibular or proprioceptive loss using posturographic analysis of stance tasks. Clin Neurophysiol 2008;119:2338–46. doi: 10.1016/j.clinph.2008.07.221 18782677 10.1016/j.clinph.2008.07.221

[3] Bloem BR, Allum JH, Carpenter MG, Honegger F. Is lower leg proprioception essential for triggering human automatic postural responses? Exp Brain Res 2000;130:375–91. doi: 10.1007/s002219900259 10706436 10.1007/s002219900259

[4] Inglis JT, Horak FB, Shupert CL, Jones Rycewicz C. The importance of somatosensory information in triggering and scaling automatic postural responses in humans. Exp Brain Res 1994;101:159–64. 7843295 10.1007/BF00243226

[5] Allum JH, Bloem BR, Carpenter MG, Honegger F. Differential diagnosis of proprioceptive and vestibular deficits using dynamic support-surface posturography. Gait Posture 2001;14:217–26. 11600325 10.1016/s0966-6362(01)00142-4

[6] Bloem BR, Allum JH, Carpenter MG, Verschuuren J, Honegger F. Triggering of balance corrections and compensatory strategies in a patient with total leg proprioceptive loss. Exp Brain Res 2002;142:91–107. doi: 10.1007/s00221-001-0926-3 11797087 10.1007/s00221-001-0926-3

[7] Horak FB. Postural orientation and equilibrium: what do we need to know about neural control of balance to prevent falls? Age Ageing 2006;35:ii7–ii11. doi: 10.1093/ageing/afl077 16926210 10.1093/ageing/afl077

[8] Jeka J, Oie K, Schöner G, Dijkstra T, Henson E. Position and velocity coupling of postural sway to somatosensory drive. J Neurophysiol 1998;79:1661–74. doi: 10.1152/jn.1998.79.4.1661 9535937 10.1152/jn.1998.79.4.1661

[9] McKeon P, Hertel J. Diminished plantar cutaneous sensation and postural control. Percept Mot Skills 2007;104:56–66. doi: 10.2466/pms.104.1.56-66 17450964 10.2466/pms.104.1.56-66

[10] Simmons RW, Richardson C, Pozos R. Postural stability of diabetic patients with and without cutaneous sensory deficit in the foot. Diabetes Res Clin Pract 1997;36:153–60. 9237781 10.1016/s0168-8227(97)00044-2

[11] Simoneau GG, Ulbrecht JS, Derr JA, Becker MB, Cavanagh PR. Postural instability in patients with diabetic sensory neuropathy. Diabetes Care 1994;17:1411–21. 7882810 10.2337/diacare.17.12.1411

[12] van Deursen RW, Simoneau GG. Foot and ankle sensory neuropathy, proprioception, and postural stability. J Orthop Sports Phys Ther 1999;29:718–26. doi: 10.2519/jospt.1999.29.12.718 10612069 10.2519/jospt.1999.29.12.718

[13] Richardson JK, Ching C, Hurvitz EA. The relationship between electromyographically documented peripheral neuropathy and falls. J Am Geriatr Soc 1992;40:1008–12. 1328346 10.1111/j.1532-5415.1992.tb04477.x

[14] Hanewinckel R, van Oijen M, Ikram MA, van Doorn P. The epidemiology and risk factors of chronic polyneuropathy. Eur J Epidemiol 2016;31:5–20. doi: 10.1007/s10654-015-0094-6 26700499 10.1007/s10654-015-0094-6 PMC4756033

[15] The Italian General Practitioner Study Group. Chronic symmetric symptomatic polyneuropathy in the elderly: a field screening investigation in two Italian regions. I. Prevalence and general characteristics of the sample. Italian General Practitioner Study Group (IGPSG). Neurology 1995;45:1832–6. 7477977 10.1212/wnl.45.10.1832

[16] Baldereschi M, Inzitari M, Di Carlo A, Farchi G, Scafato E, Inzitari D. Epidemiology of distal symmetrical neuropathies in the Italian elderly. Neurology 2007;68:1460–7. doi: 10.1212/01.wnl.0000260606.36443.29 17470747 10.1212/01.wnl.0000260606.36443.29

[17] Callaghan B, Price R, Feldman E. Distal Symmetric Polyneuropathy: A Review. JAMA 2015;314:2172–81. doi: 10.1001/jama.2015.13611 26599185 10.1001/jama.2015.13611 PMC5125083

[18] Hoffman EM, Staff N, Robb J, St Sauver J, Dyck P, Klein C. Impairments and comorbidities of polyneuropathy revealed by population-based analyses. Neurology 2015;84:1644–51. doi: 10.1212/WNL.0000000000001492 25832668 10.1212/WNL.0000000000001492 PMC4409579

[19] Martyn CN, Hughes RA. Epidemiology of peripheral neuropathy. J Neurol Neurosurg Psychiatry 1997;62:310–8. 9120441 10.1136/jnnp.62.4.310 PMC1074084

[20] Bharucha NE, Bharucha AE, Bharucha EP. Prevalence of peripheral neuropathy in the Parsi community of Bombay. Neurology 1991;41:1315–7. 1650932 10.1212/wnl.41.8.1315

[21] Watson J, Dyck PJB. Peripheral Neuropathy: A Practical Approach to Diagnosis and Symptom Management. Mayo Clin Proc 2015;90:940–51. doi: 10.1016/j.mayocp.2015.05.004 26141332 10.1016/j.mayocp.2015.05.004

[22] Callaghan B, Kerber K, Lisabeth L, Morgenstern L, Longoria R, Rodgers A, et al Role of neurologists and diagnostic tests on the management of distal symmetric polyneuropathy. JAMA Neurol 2014;71:1143–9. doi: 10.1001/jamaneurol.2014.1279 25048157 10.1001/jamaneurol.2014.1279 PMC4266395

[23] Lubec D, Müllbacher W, Finsterer J, Mamoli B. Diagnostic work-up in peripheral neuropathy: an analysis of 171 cases. Postgrad Med J 1999;75:723–7. 10567598 10.1136/pgmj.75.890.723 PMC1741419

[24] Vallat J-M, Sommer C, Magy L. Chronic inflammatory demyelinating polyradiculoneuropathy: diagnostic and therapeutic challenges for a treatable condition. Lancet Neurol 2010;9:402–12. doi: 10.1016/S1474-4422(10)70041-7 20298964 10.1016/S1474-4422(10)70041-7

[25] Mahdi Rogers M, Hughes RAC. Epidemiology of chronic inflammatory neuropathies in southeast England. Eur J Neurol 2014;21:28–33. doi: 10.1111/ene.12190 23679015 10.1111/ene.12190

[26] England JD, Gronseth GS, Franklin G, Carter GT, Kinsella LJ, Cohen JA, et al Practice Parameter: evaluation of distal symmetric polyneuropathy: role of laboratory and genetic testing (an evidence-based review). Report of the American Academy of Neurology, American Association of Neuromuscular and Electrodiagnostic Medicine, and American Academy of Physical Medicine and Rehabilitation. Neurology 2009;72:185–92. doi: 10.1212/01.wnl.0000336370.51010.a1 19056666 10.1212/01.wnl.0000336370.51010.a1

[27] Van den Bergh PYK, Hadden RDM, Bouche P, Cornblath DR, Hahn A, Illa I, et al European Federation of Neurological Societies/Peripheral Nerve Society guideline on management of chronic inflammatory demyelinating polyradiculoneuropathy: report of a joint task force of the European Federation of Neurological Societies and the Peripheral Nerve Society—first revision. Eur J Neurol 2010;17:356–63. doi: 10.1111/j.1468-1331.2009.02930.x 20456730 10.1111/j.1468-1331.2009.02930.x

[28] Hegeman J, Shapkova EY, Honegger F, Allum JHJ. Effect of age and height on trunk sway during stance and gait. J Vestib Res 2007;17:75–87. 18413900

[29] Goutier KMT, Jansen SL, Horlings CGC, Küng UM, Allum JHJ. The influence of walking speed and gender on trunk sway for the healthy young and older adults. Age Ageing 2010;39:647–50. doi: 10.1093/ageing/afq066 20558480 10.1093/ageing/afq066

[30] Allum JH, Carpenter MG. A speedy solution for balance and gait analysis: angular velocity measured at the centre of body mass. Curr Opin Neurol 2005;18:15–21. 15655397 10.1097/00019052-200502000-00005

[31] Bril V. NIS-LL: The Primary Measurement Scale for Clinical Trial Endpoints in Diabetic Peripheral Neuropathy. Eur Neurol 1999;41(suppl 1):8–13. 10023123 10.1159/000052074

[32] Gelb DJ. The Neurologic Examination Introduction to Clinical Neurology. Woburn, MA: Butterworth Heinemann; 2000.

[33] Dyck PJ, Kratz KM, Lehman KA, Karnes JL, Melton LJ, O'Brien PC, et al The Rochester Diabetic Neuropathy Study: design, criteria for types of neuropathy, selection bias, and reproducibility of neuropathic tests. Neurology 1991;41:799–807. 2046920 10.1212/wnl.41.6.799

[34] Jacobson GP, Newman CW. The development of the Dizziness Handicap Inventory. Arch Otolaryngol Head Neck Surg 1990;116:424–7. 2317323 10.1001/archotol.1990.01870040046011

[35] Horlings CGC, Küng UM, van Engelen BGM, Voermans NC, Hengstman GJD, van der Kooi AJ, et al Balance control in patients with distal versus proximal muscle weakness. Neuroscience 2009;164:1876–86. doi: 10.1016/j.neuroscience.2009.09.063 19796669 10.1016/j.neuroscience.2009.09.063

[36] de Hoon EW, Allum JH, Carpenter MG, Salis C, Bloem BR, Conzelmann M, et al Quantitative assessment of the stops walking while talking test in the elderly1. Arch Phys Med Rehabil 2003;84:838–42. 12808535 10.1016/s0003-9993(02)04951-1

[37] Bischoff Ferrari HA, Conzelmann M, Stähelin HB, Dick W, Carpenter MG, Adkin AL, et al Is fall prevention by vitamin D mediated by a change in postural or dynamic balance? Osteoporos Int 2006;17:656–63. doi: 10.1007/s00198-005-0030-9 16508700 10.1007/s00198-005-0030-9

[38] Adkin AL, Bloem BR, Allum JHJ. Trunk sway measurements during stance and gait tasks in Parkinson's disease. Gait Posture 2005;22:240–9. 16278966 10.1016/j.gaitpost.2004.09.009

[39] Corporaal SH, Gensicke H, Kuhle J, Kappos L, Allum JH, Yaldizli O. Balance control in multiple sclerosis: correlations of trunk sway during stance and gait tests with disease severity. Gait Posture 2013;37:55–60. doi: 10.1016/j.gaitpost.2012.05.025 22874664 10.1016/j.gaitpost.2012.05.025

[40] Fanchamps MH, Gensicke H, Kuhle J, Kappos L, Allum JH, Yaldizli O. Screening for balance disorders in mildly affected multiple sclerosis patients. J Neurol 2012;259:1413–9. doi: 10.1007/s00415-011-6366-5 22186852 10.1007/s00415-011-6366-5

[41] Allum JHJ, Honegger F. Recovery times of stance and gait balance control after an acute unilateral peripheral vestibular deficit. J Vestib Res 2016;25:219–31. doi: 10.3233/VES-150561 26890423 10.3233/VES-150561

[42] Findling O, Sellner J, Meier N, Allum JH, Vibert D, Lienert C, et al Trunk sway in mildly disabled multiple sclerosis patients with and without balance impairment. Exp Brain Res 2011;213:363–70. doi: 10.1007/s00221-011-2795-8 21773798 10.1007/s00221-011-2795-8

[43] Allum JH, Adkin AL. Improvements in trunk sway observed for stance and gait tasks during recovery from an acute unilateral peripheral vestibular deficit. Audiol Neurootol 2003;8:286–302. doi: 10.1159/000071999 12904683 10.1159/000071999

[44] Kulkarni G, Mahadevan A, Taly A, Nalini A, Shankar SK. Sural nerve biopsy in chronic inflammatory demyelinating polyneuropathy: are supportive pathologic criteria useful in diagnosis? Neurol India 2010;58:542–8. doi: 10.4103/0028-3886.68673 20739789 10.4103/0028-3886.68673

[45] Oh SJ, Joy JL, Kuruoglu R. "Chronic sensory demyelinating neuropathy": chronic inflammatory demyelinating polyneuropathy presenting as a pure sensory neuropathy. J Neurol Neurosurg Psychiatry 1992;55:677–80. 1326601 10.1136/jnnp.55.8.677 PMC489203

[46] Abboud RJ, Rowley DI, Newton RW. Lower limb muscle dysfunction may contribute to foot ulceration in diabetic patients. Clin Biomech (Bristol, Avon) 2000;15:37–45. 10.1016/s0268-0033(99)00038-8 10590343

[47] Mueller MJ, Minor SD, Sahrmann SA, Schaaf JA, Strube MJ. Differences in the gait characteristics of patients with diabetes and peripheral neuropathy compared with age-matched controls. Phys Ther 1994;74:299–308; discussion 9. 8140143 10.1093/ptj/74.4.299

[48] Allum JHJ, Scheltinga A, Honegger F. The effect of peripheral vestibular recovery on improvements in vestibular-ocular reflexes and balance control after acute unilateral vestibular loss. Otol Neurotol 2017a;in press. 10.1097/MAO.0000000000001477 29135873

